# Maintenance Treatment with 5-Azacitidine in Patients with Acute Myeloblastic Leukemia Ineligible for Intensive Treatment and with Response After Induction Chemotherapy: A Phase II Clinical Trial

**DOI:** 10.3390/cancers17162678

**Published:** 2025-08-18

**Authors:** Alfonso Fernández Fernández, María García Fortes, Mar Tormo Díaz, María Luz Juan Marco, Rebeca Cuello García, Adolfo de La Fuente, Josefina Serrano López, Mª Ángeles Medina Pérez, Miguel Ángel Sánchez Chaparro, Regina García Delgado

**Affiliations:** 1 Hospital Universitario Virgen de la Victoria, 29010 Málaga, Spain; 2 Hospital Clínico Universitario de Valencia, 46010 Valencia, Spain; 3 Hospital Universitario Doctor Peset, 46017 Valencia, Spain; 4 MD Anderson Cancer Center Madrid, 28033 Madrid, Spain; 5 Hospital Universitario Reina Sofía de Córdoba, 14004 Córdoba, Spain; 6 Hospital Universitario Costal del Sol, Marbella, 29603 Málaga, Spain

**Keywords:** acute myeloid leukemia, 5-azacitidine, maintenance therapy, complete remission

## Abstract

**Background/Objectives**: After first-line treatment, elderly patients with acute myeloid leukemia (AML) often become unfit to continue intensive chemotherapy despite having achieved a response. This trial aimed to determine the efficacy of maintenance treatment with azacitidine in AML patients who are ineligible to continue intensive treatment after remission. **Methods**: A single-arm, multicenter, phase II clinical trial (EudraCT: 2010-020432-18) including patients with AML with complete (CR) or partial remission (PR) after one or two cycles of intensive induction chemotherapy and ineligible to continue intensive treatment was conducted. Efficacy was measured as the response rate, progression-free survival (PFS), and overall survival (OS), and was assessed in the intention-to-treat (ITT) population (all patients). Quality of life was also assessed. **Results**: Thirty-two patients were included, with a mean age of 73.3 years (SD 3.8) (53.1% male); sixteen patients (50.0%) reached the sixth treatment cycle. The best response was CR in 11 patients (68.8%) and PR in 2 patients (12.5%) at cycle six, and CR in 15 patients (46.9%) and PR in 5 patients (15.6%) overall. The median PFS was 6.7 months (95% CI 3.1–8.7), and OS was 11.5 months (95% CI 6.6–15.9). The daily function (*p* = 0.0296), cognitive function (*p* = 0.0412), and social function (*p* = 0.0275) scales of the EORTC QLQ-C30 questionnaire significantly improved. Thirty-one patients (96.9%) experienced adverse events; six (1.9%) were serious. **Conclusions**: Azacitidine is a safe and well-tolerated maintenance treatment option for AML patients unfit for intensive therapy following a response to induction, although the single-arm phase II design precludes direct causal inference. Patients achieved promising results regarding PFS and remission rates, with improved quality of life.

## Simple Summary

Elderly patients with acute myeloid leukemia (AML) are frequently unfit to continue intensive chemotherapy despite having achieved a response to first-line induction chemotherapy. Treatments prolonging the remission time with minimal non-relapse mortality risk are an unmet need in this patient population. This clinical trial aimed to determine the efficacy of azacitidine as a maintenance treatment for patients with AML who are unfit to continue intensive chemotherapy. We found favorable outcomes of azacitidine in our population of 32 patients with a mean age of 73.3 years (SD 3.8), with increased PFS and OS, and 33.3% of the patients with a previous partial remission achieved complete remission. Moreover, patients maintained an ECOG score of 0, with significantly improved quality of life in daily, cognitive, and social function scales. Azacitidine is a safe and well-tolerated maintenance treatment option for AML patients who are unfit for intensive therapy following a response to induction therapy.

## 1. Introduction

Acute myeloid leukemia (AML) is the most common leukemia among adults, with a median age of 68 years at diagnosis [1]. AML is characterized by uncontrolled proliferation of immature hematopoietic cells [2] and impaired production of normal blood cells [3], causing bone marrow failure and leading to leukopenia, anemia, and thrombocytopenia [4].

The etiology of AML is heterogeneous and, in most cases, remains unknown [4]. The most common risk factors are myelodysplastic syndrome [5] or exposure to DNA-damaging agents [4]. The incidence of AML varies with gender and race and increases by approximately 2.2% per year, with more than 30% of patients being 78 years or older at diagnosis [4,6]. The age-adjusted incidence is 2.0 per 100,000 person–years in the population ≤ 65 years and increases to 20.1 per 100,000 person–years in the population ≥ 65 years [1]. Patient prognosis worsens with age, with reported 5-year survival rates of 20–30% in patients aged ≤60 years and 10–15% in those ≥60 years [7].

The World Health Organization (WHO), in 2022, re-envisioned the AML classification based on genetic abnormalities, differentiation, cytogenetic abnormalities, and somatic mutations to emphasize how this disease is understood and managed [8]. In AML, patient prognosis is based on AML genetics, patient baseline status, and minimal residual disease (MRD), an important biomarker for prognostic, predictive, monitoring, and efficacy–response assessments [8,9]. In this context, comprehensive functional assessment, an evolutionary variable that effectively integrates all relevant prognostic data to better inform patient care, is a useful tool for a thorough assessment of AML patients [9,10].

The mainstay first-line treatment for eligible or fit AML patients consists of induction treatment with intensive chemotherapy and consolidation, followed or not by allogeneic hematopoietic cell transplantation (allo-HCT) [11,12,13]. For unfit patients, the first-line treatment consists of non-intensive treatment with azacitidine (AZA) in monotherapy or in combination with venetoclax; for frail patients, palliative care alone is recommended [11]. Nevertheless, the most advisable option for any of these patients would be to include them in a clinical trial.

In elderly patients, the response rates to intensive chemotherapy range from 30% to 50%, which is lower than in younger patients (80% to 90% in patients < 55 years). Moreover, hospitalizations are longer, and early mortality associated with treatment is around 24% in patients > 65 years [14]. Furthermore, after first-line treatment, elderly patients often become unfit to continue intensive chemotherapy despite having achieved a response. For these patients, a suitable alternative to intensive chemotherapy may be maintenance therapy with AZA alone to control the undetectable but residual load of leukemic cells and improve overall survival [15,16]. In 2008, the European Medicines Agency approved AZA for the treatment of patients with AML > 64 years and 20% to 30% bone marrow blasts, ineligible for hematopoietic stem-cell transplantation [17].

This single-arm phase II clinical trial aimed to determine the efficacy of AZA as a maintenance treatment for AML patients unfit to continue intensive therapy after a response to induction treatment with intensive chemotherapy. Secondarily, we aimed to assess the impact of maintenance treatment with AZA on patients’ quality of life and its safety profile.

## 2. Materials and Methods

### 2.1. Study Design and Participants

This was a single-arm, multicenter, monitored phase II clinical trial. The study was carried out from January 2012 to January 2019 in patients with AML and complete remission (CR) or partial remission (PR) after 1 or 2 cycles of induction chemotherapy (cytarabine 100 mg/m^2^ during 5 days and idarubicin 10 mg/m^2^ during 2 days), ineligible to continue intensive treatment. All eligible patients meeting the inclusion criteria were enrolled consecutively at each participating site during the recruitment period.

Patients received maintenance treatment with AZA for six cycles (treatment period) at eight centers across Spain. Efficacy and safety assessments were performed on day 1 of each treatment cycle until the end. After the sixth cycle, patients without disease progression (DP) continued treatment. Patients with DP in any cycle or who decided not to continue for any other reason stopped the treatment definitively and underwent the end-of-study visit, during which the evolution of unresolved toxicities was documented. In the follow-up visits, survival was recorded every 12 weeks through telephone calls until death. Patients with no DP at the end-of-study visit were followed until DP was documented.

The protocol was approved by the Autonomous Committee for Clinical Trials of Andalusia (Seville, Spain). The study was carried out following the ethical principles of the Declaration of Helsinki (Seoul version, October 2008) and the Good Clinical Practices (GCP) of the International Council for Harmonization (ICH). Before any procedure was performed, the patients’ informed consent was obtained. This trial (code LAMAN-01AN) was registered at the European Union Drug Regulating Authorities Clinical Trials Database (EudraCT: 2010-020432-18).

### 2.2. Selection Criteria

This study included adult patients (≥18 years), unfit for intensive chemotherapy, with untreated de novo AML, secondary AML, history of myelodysplastic syndrome (MDS), and with CR or PR after 1–2 cycles of the induction treatment. Patients included had to have an Eastern Cooperative Oncologic Group (ECOG) score of ≤2 or an ECOG score of 3 due to hematologic disease. Additional inclusion criteria were suitable liver function (AST and ALT 2.5 times the upper limit of normal (ULN), total bilirubin < 1.5 times the ULN) and renal function with creatinine clearance > 60 mL/min or serum creatinine < 1.5 times the ULN.

Exclusion criteria were hypersensitivity to AZA, previous treatment for AML, refractory or relapsed AML, severe or unstable concomitant disease or severe active infection that may contraindicate initiation of chemotherapy, life expectancy < 1 month, and psychiatric illness or mental disorder that may compromise compliance with treatment. Additional exclusion criteria were participation in another clinical drug trial (in the previous 30 days), radiotherapy, chemotherapy, or cytotoxic treatment for conditions other than AML (four weeks before study entry), and active neoplasm other than AML. Women planning to become pregnant, pregnant, lactating, or those not willing to use effective contraception, and patients with legal incapacity or limited legal capacity were excluded.

### 2.3. Treatment

The first cycle with AZA depended on each patient’s remission degree after induction therapy [18]. Patients with CR were administered 50 mg/m^2^ of AZA daily for 5 days every 28 days, and patients with PR were given two cycles of AZA 75 mg/m^2^ daily for 5 days every 28 days. The following cycles had the same scheme: 50 mg/m^2^ of AZA daily for 5 days every 28 days for all patients. The dose adjustment of AZA treatment in each cycle was based on the blood count before AZA administration, bone marrow cellularity, and the recovery time (Supplementary Table S1). It was calculated according to the patient’s body weight (only recalculated if body weight changed ≥ 10% from baseline). After a dose reduction due to toxicity, there was no increase during the study period.

### 2.4. Efficacy Endpoints and Variables

Baseline data included demographic (sex and age) and clinical characteristics (bone marrow cytometry and morphology, ECOG, type of cytopenia, comorbidities, and response to previous induction treatment), which were collected during the four weeks before starting treatment with AZA.

The treatment efficacy was evaluated in the sixth treatment cycle. The primary efficacy endpoint was the rate of treatment response, defined as patients with previous PR and CR at the end of the treatment (6 cycles with AZA), which included CR, PR, and DP, according to the International Working Group (IWG) 2003 response criteria classification [19] (Supplementary Table S2). Other variables assessed were the best response obtained during treatment, the progression-free survival (PFS) (time elapsed from the administration of the first dose to the date of DP), death due to any cause or early treatment discontinuation, and the overall survival (OS) (time from the first dose to the date of death due to any cause).

The EORTC QLQ-C30 quality of life questionnaire was used to assess the secondary efficacy objective (i.e., the impact of the treatment on patients’ quality of life); it is a specific cancer questionnaire made up of 30 questions or items and validated in more than 80 languages [20]. The EORTC QLQ-C30 was registered after cycles 1, 4, 6, and 12.

On day one of each cycle, variables assessed and data collected were concomitant medication, adverse events (AEs), ECOG functional status assessment, a physical examination with vital signs, weight, and body surface area, peripheral blood for the hematological and biochemical study, pregnancy tests in blood or urine in women with the possibility of conception, and bone marrow aspirate.

### 2.5. Safety Assessments

Treatment safety and tolerability were also evaluated in the first six months to take corrective measures regarding the treatment dose and the end-of-study visit. All types of AEs and reactions were recorded, including the date of appearance, duration, and resolution, and the measures adopted in each case. AEs were classified according to intensity on a 1 to 5 scale following the Common Terminology Criteria for Adverse Events (CTCAE version 4.03) of the National Cancer Institute (NCI) [21].

### 2.6. Statistical Analysis

Based on the previously reported 50% survival rate of AML patients receiving maintenance treatment [22], and considering a 10% dropout rate, data from at least 30 patients would be necessary to assess the primary endpoint with a 95% one-sided confidence interval (CI) and a 15% precision.

Categorical variables were presented as frequencies and percentages, and continuous variables were presented as mean, standard deviation (SD), median, 25% and 75% (Q1 and Q3), and extreme values (minimum and maximum). Categorical variables were compared using the Chi-square test or Fisher’s exact test. We used the T-Test, ANOVA, non-parametric Wilcoxon, or Kruskal–Wallis tests to compare continuous and categorical variables. The Pearson or Spearman correlation was used to assess the relationship between two continuous variables.

All analyses were performed on the intention-to-treat (ITT) population, including all patients in the study.

For survival analyses, survival curves were estimated using the Kaplan–Meier method. For the PFS and OS analyses, patients who discontinued without progression or follow-up information on their death were considered as last-minute censored observations when they did not progress or were still alive. In patients without the date of death, day 15 was imputed to calculate PFS and OS. For other missing data, no imputation was carried out. The analysis was performed on available cases at each visit. The progressive reduction in sample size throughout the study visits reflects treatment discontinuation due to progression, toxicity, or death, as expected in this patient population. Reasons for permanent withdrawal (disease progression vs. adverse events) and the treatment cycle at last dose were summarized; no pre-planned subgroup analyses were performed. Regarding the impact on QoL, we analyzed changes in questionnaire scores across treatment visits using an adjusted mixed-linear model.

All statistical analyses were performed with the statistical package SAS^®^ System version 9.4. The significance level used for all tests was set at a two-sided α < 0.05, and no adjustments for multiple comparisons or corrections for multiplicity were carried out.

## 3. Results

### 3.1. Characteristics of the Study Population

**Figure 1:**
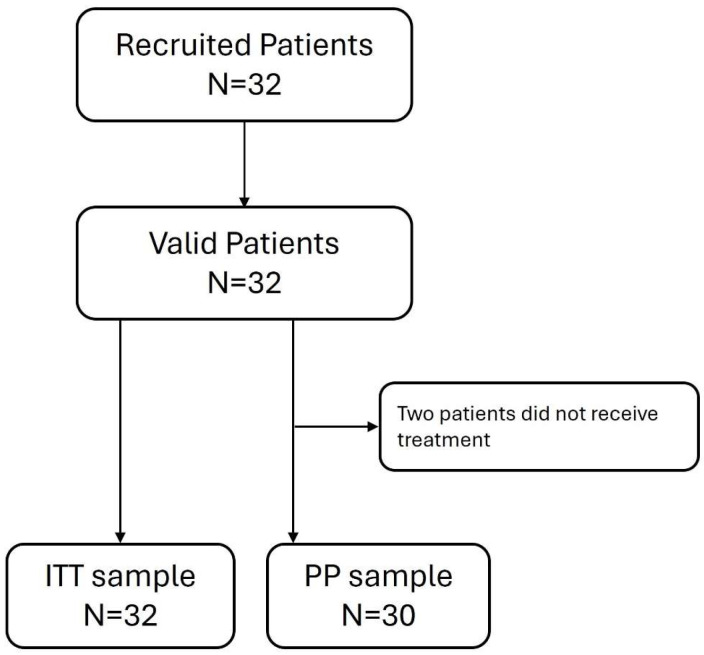
Study flowchart. ITT, intention to treat; PP, per protocol [The two patients who did not receive treatment were excluded because of a major protocol deviation].

Thirty-two patients were included in this study with a mean age of 73.3 years (SD 3.8); 53.1% (*n* = 17) were male. Of them, two patients did not receive any treatment cycle and were excluded, resulting in an ITT (intention-to-treat) sample of *n* = 32 and a PP (per protocol) sample of *n* = 30 (Figure 1). The study sample was evenly distributed according to sex. Half of the patients had an ECOG = 0, and most patients (61.3%) had CR to induction chemotherapy (Table 1). The patients’ demographic and baseline clinical characteristics are summarized in Table 1; the clinical findings/comorbidities are detailed in Supplementary Table S3. Cytogenetic data were available for 31 of the 32 patients (in the sample from 1 of the patients, no mitotic cells were observed). One patient (3.2%) exhibited inv(16), corresponding to a favorable-risk karyotype. A total of 11 patients (34.4%) had AML associated with prior myelodysplastic syndrome (MDS), and the remainder had either normal or unclassifiable cytogenetics.

The median follow-up was 11.8 months (Q1 6.0, Q3 18.5; range 1–48 months), with a mean treatment duration of 5.0 months (Q1 2.2, Q3 7.3; range 0–21).

### 3.2. Primary Efficacy Outcome: Treatment Response

A total of 16 patients (50.0%) reached the sixth treatment cycle: 11 (68.8%) with CR, 2 (12.5%) with PR, 1 (6.3%) with relative resistance, and 2 (12.5%) progressed in that cycle. Six patients with PR after induction therapy were still on treatment at cycle 6; two (33.3%) achieved CR at the sixth cycle with AZA, while four (66.7%) did not improve. The PP analysis yielded similar results: After six treatment cycles with AZA, the best response obtained in cycle six was CR in 11 patients (68.8%) and PR in 2 patients (12.5%), and the best response obtained during the study was CR in 15 patients (46.9%) and PR in 5 patients (15.6%). Two patients (12.5%) had DP at cycle 6, and ten patients (31.3%) had DP throughout the AZA treatment.

**Figure 2:**
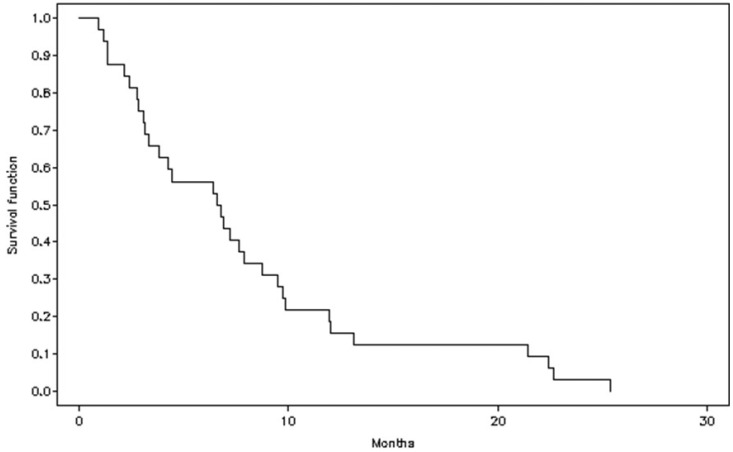
Estimation of progression-free survival (PFS).

**Figure 3:**
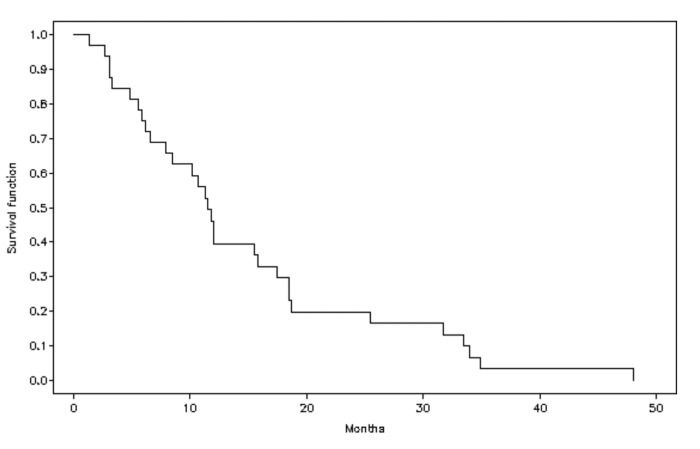
Estimation of overall survival (OS).

The median PFS was 6.7 months (95% CI 3.1–8.7) (Figure 2), and the median OS was 11.5 months (95% CI 6.6–15.9) (Q1, 6.0 [95% CI 3.1–10.2]; Q3 18.5 [95% CI 12.1–33.5]) (Figure 3).

### 3.3. Impact of AZA Treatment on Quality of Life (QoL)

**Figure 4:**
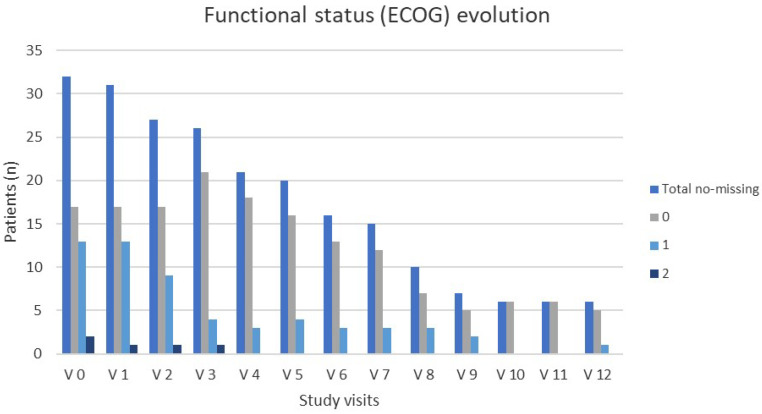
ECOG functional status (0, 1, and 2) of study patients throughout the study. Columns represent the number of patients.

The impact of treatment with AZA on patients’ overall QoL showed certain improvement, although this was not statistically significant (Table 2). Significant improvements were observed in the daily function (*p* = 0.0296), cognitive function (*p* = 0.0412), and social function (*p* = 0.0275) scales, but not in the rest of the functions or the symptomatology scales. The evolution of patients’ ECOG functional status throughout the study is shown in Figure 4. Although the proportion of patients with an ECOG score of 0 increased over time, this was not due to true functional improvement in most cases. Rather, patients with poorer baseline PS discontinued the study early due to death or disease progression and were no longer represented in later visits. Among patients who remained on treatment, ECOG performance status remained generally stable throughout the study period.

### 3.4. Safety Outcomes

A total of 31 patients (96.9%) experienced 308 AEs, of which 6 were SAEs in 5 patients; 118 AEs (38.3%) were treatment-related (Table 3). Most AEs were G1-G2 (mild and moderate) (n = 228). No SAEs related to treatment were life-threatening or caused death, and only one SAE was related to AZA.

Nine patients (28.1%) experienced neutropenia and required dose modifications. Temporary discontinuation of AZA was required in 19 (59.4%) patients due to AEs, including 12 cases of neutropenia, 4 cases of thrombocytopenia, 1 case of febrile neutropenia, 1 case of abdominal pain, and 1 case of infection caused by a medical device. In 11 patients (34.4%), AZA was permanently discontinued due to neutropenia in 4 cases, thrombocytopenia in 4 cases, and febrile neutropenia, anemia, and rash in 1 case each.

The most frequent AEs were neutropenia (56.3%), anemia (34.4%), and thrombocytopenia (31.3%), which were in line with those described in the AZA datasheet [23]. A complete list of AEs classified by System Organ Class (SOC) and Preferred Term (PT) is provided in Supplementary Table S4.

## 4. Discussion

Intensive chemotherapy, in combination with allogeneic hematopoietic cell transplantation and supportive care, can induce long-term remissions in approximately 50% of all AML patients eligible for intensive treatment [24]. However, intensive chemotherapy is associated with toxicity, particularly in older adults. These toxic effects include physical deconditioning, declines in quality of life (QOL), increased fatigue, and early mortality [25]. As we commented above, many fit elderly adults have unsatisfactory treatment outcomes. Despite having a response to induction with intensive chemotherapy, in a new evaluation, many of them are unfit for continuing intensive treatment. These findings have promoted studies on less toxic and more efficient agents. Nevertheless, prolonging the remission time with minimal non-relapse mortality risk after induction with intensive chemotherapy remains an unmet need for patients who become unfit to continue intensive chemotherapy.

The main goals of maintenance therapy following the achievement of post-consolidation CR are to prolong response and improve OS, particularly in patients who are ineligible for stem allo-HCT or unable to receive additional intensive therapy due to age, frailty, comorbidities, prior toxicities, or the potential for worsening quality of life [26]. The initial studies that analyzed the efficacy of maintenance therapy in AML were conducted in the 1980s. The results obtained, due to different populations and different induction and consolidation strategies, make it challenging to ascertain whether maintenance therapy based on chemotherapy strategies (oral, subcutaneous, or intravenous) offers the most efficacious outcomes for AML patients.

In our phase II clinical trial with patients unfit to continue intensive therapy after a response to induction, the results demonstrated favorable outcomes after six cycles of maintenance treatment with AZA. As evidenced by other studies that also employed injectable AZA [27,28,29], or even those that utilized oral AZA [30], as a maintenance therapy, our patients also showed increased PFS. Furthermore, patients had an improved OS, a benefit previously observed only after maintenance therapy with oral AZA [30]. Additionally, 33.3% of patients with PR before maintenance therapy achieved CR. The greatest improvement in ECOG score, with more patients achieving an ECOG score of 0, was observed between visits three and five, and most patients who remained alive maintained an ECOG score of 0. Our PFS and OS results were consistent with those obtained in the AZA arm of the phase III study [31], which was used to obtain approval for AZA in 2008 as a first-line treatment for AML patients older than 65 years who were unfit for allo-HCT [17]. Our patients’ QoL improved significantly in the daily, cognitive, and social function scales. Despite a 16-point rise, the global health status scale did not reach statistical significance (*p* = 0.0712), likely owing to sample attrition and baseline heterogeneity; by contrast, improvements in daily (+24.7), cognitive (+17.5), and social (+25.1) functions exceeded the 10-point threshold for clinical relevance. The absence of a significant change in global QoL probably reflects the lower sensitivity of this composite scale, small numbers at later visits, and inter-patient variability. Importantly, clinically meaningful (>10-point) gains were recorded in daily, cognitive, and social domains and were sustained up to 12 months in those who continued therapy.

Previous studies have shown a potential clinical benefit of AZA as a single-agent maintenance therapy or as part of combined regimens. In 2015, the UK NCRI AML16 trial showed an increased 5-year OS rate in MRD-negative patients randomized to receive maintenance treatment with AZA compared to observation (40% vs. 13%) [28]. The phase III QUAZAR AML-001 trial of oral azacitidine (CC-486) in patients ≥ 55 years in first CR/CRi reported a median relapse-free survival of 10.2 months and a median overall survival of 24.7 months, versus 4.8 and 14.8 months with placebo, respectively [30]. In the randomized HOVON-97 study, subcutaneous AZA (50 mg m^2^ × 5 days/28 days) given to patients ≥ 60 years in CR/CRi yielded a median disease-free survival of 15.9 months compared with 10.3 months for observation, without a significant OS advantage (84% vs. 70% at 12 months). Against these benchmarks, our older and less-fit LAMAN cohort (mean age 73 years; 38% in partial remission) showed a median PFS of 6.7 months, a median OS of 11.5 months, and a complete remission rate of 46.9% (68.8% among patients who completed cycle 6), highlighting that parenteral AZA maintenance is associated with clinically meaningful disease control even in frail patients, although causality cannot be inferred from this single-arm phase II design [27]. A notable distinction of our study compared to prior maintenance trials such as QUAZAR and HOVON97 is the inclusion of patients in partial remission (PR) after induction. While most previous trials only enrolled patients in complete remission, our findings suggest that azacitidine may provide clinical benefit even in those who have not achieved full remission. A recent single-center, single-arm, phase 2 trial, in patients in complete remission or complete remission with incomplete blood count recovery and not immediately eligible for hematopoietic stem-cell transplantation (HSCT), has demonstrated that a low-dose AZA plus venetoclax is a feasible maintenance strategy in AML following intensive and low-intensity induction [32]. This previous evidence supports the favorable outcomes observed in this study, suggesting that maintenance treatment with AZA would be an appropriate option for patients ineligible to continue intensive treatment after a response to induction therapy, likely fulfilling the unmet need to prolong the remission time and OS.

The results from our study should be interpreted in the context of limitations associated with its design and size. Despite being a clinical trial, the lack of a comparative treatment arm precluded establishing side-by-side comparisons with other standard maintenance treatments. Regarding the study’s size, a larger sample size with more patients may have compensated for the sample attrition, allowing for more robust conclusions regarding PFS and OS, and additional subgroup analyses based on patients’ clinical characteristics. Because salvage treatments after study withdrawal and on-treatment disease status at AE-driven discontinuation were not prospectively recorded, a more granular characterization of early discontinuation was not possible; this should be addressed in future trials. Furthermore, at the time this study was performed, oral AZA and venetoclax treatments were not available. Specifically, oral administration of AZA eliminates parenteral treatment and its associated side effects, likely resulting in improved patient outcomes compared to parenteral AZA. Despite these limitations, this study provides valuable data regarding the efficacy and safety of maintenance treatment with AZA in a population of AML patients unfit to continue intensive therapy after induction therapy. The results indicate that this treatment is a suitable option for this population.

The absence of a randomized comparator arm restricts internal validity. While single-arm designs are accepted in early-phase research, they do not permit definitive efficacy claims.

## 5. Conclusions

Azacitidine was a safe and well-tolerated maintenance treatment option for AML patients who were unfit for intensive therapy following a response to induction therapy. Patients achieved promising results in PFS and CR, and a significant improvement in some daily function scales was observed after six cycles of treatment. However, because the present study lacked a control arm, these outcomes cannot be definitively attributed to AZA. The results should therefore be interpreted with caution and serve primarily to inform the design of future randomized trials.

## Figures and Tables

**Table 1:** Baseline demographic and clinical characteristics of study patients; N = 32.

Demographics (*n* = 32)	
**Sex**, *n (%)*	
Male	17 (53.1)
Female	15 (46.9)
**Age (years)**, *mean (SD)*	73.3 (3.8)
**Clinical characteristics (*n* = 32)**	
ECOG scale, *n (%)*	
0	17 (53.1)
1	13 (40.6)
2	2 (6.3)
**Flow cytometry (bone marrow)**, *mean (SD)*	
Blasts	51.4 (31.7)
**Bone marrow morphology**, *n (%)*	
Hypercellular	16 (50.0)
Hypocellular	9 (28.1)
Normocellular	7 (21.9)
**Cytopenia at baseline**, *n (%)*	
Anemia	6 (18.8)
Leucopenia	4 (12.5)
Neutropenia	4 (12.5)
Thrombocytopenia	2 (6.3)
**Response to induction chemotherapy**, *n (%)*	
Complete remission	19 (61.3)
Partial remission	12 (38.7)

Abbreviations: ECOG, Eastern Cooperative Oncology Group; SD, standard deviation.

**Table 2:** Scores for the general and functional health scales, EORTC QLQ-Q30; mean (SD).

	N	General	PF	DF (Role)	ES	CF	SF
V 0	26	67.9 (23.8)	70.4 (27.8)	62.2 (37.6)	74.7 (29.6)	80.1 (22.6)	73.7 (25.9)
V 1	29	73.9 (22.2)	78.2 (23.3)	75.3 (32.6)	79.3 (22.9)	88.1 (18.6)	77.6 (30.3)
V 4	20	67.1 (26.0)	78.0 (18.7)	71.7 (31.6)	79.4 (20.5)	88.3 (14.4)	79.2 (29.1)
V 6	14	83.9 (13.7)	83.8 (19.9)	86.9 (24.6)	89.9 (13.9)	97.6 (6.1)	98.8 (4.5)
V 12	4	91.7 (9.6)	85.0 (3.3)	91.7 (16.7)	81.3 (12.5)	95.8 (8.3)	100.0 (0.0)
*p*-value		0.0712	0.1200	**0.0296**	0.1969	**0.0412**	**0.0275**

Abbreviations: V, visit, PF, physical function; DF, daily function; ES, emotional state; CF, cognitive function; SF, social function. V0 = baseline (pre-cycle 1); V1 = end of cycle 1 (week 4); V4 = end of cycle 4 (week 16); V6 = end of cycle 6 (week 24); V12 = end of cycle 12 (week 48). Bold font has been used to highlight values that are statistically significant.

**Table 3:** Summary of adverse events (AEs) and serious adverse events (SAEs).

		AEs	SAEs
	N-AEs N (%)-Patients	308 31 (96.9)	6 5 (15.6)
Severity *	G1	134	-
	G2	94	-
	G3	34	5
	G4	12	-
	Mild	19	-
	Moderate	11	1
	Severe	4	-

* Two severity scales were used: G1–G5 for adverse events according to the CTC (Common Toxicity Criteria) and mild/moderate/severe/life-threatening/death for other adverse events according to the Common Terminology Criteria for Adverse Events (CTCAE version 4.03) of the National Cancer Institute (NCI). No patients experienced life-threatening adverse events or death/G5 due to adverse events. Abbreviations: AE, adverse event; SAE, serious adverse event; SD, standard deviation.

## Data Availability

The datasets that support the findings of this study are available from the corresponding author upon reasonable request.

## Supplementary Materials

The following supporting information can be downloaded at https://www.mdpi.com/article/10.3390/cancers17162678/s1. Table S1: Dose adjustment of AZA treatment based on (1) blood count before the administration of AZA and (2) bone marrow cellularity and recovery time; Table S2: IWG 2006 response criteria, extracted from Komrokji et al. [19]; Table S3: Baseline findings in the clinical history/comorbidities according to the MedDRA dictionary, *n* (%); Table S4: Description of adverse events presented by System Organ Class (SOC) and Preferred Term (PT)*, *n* (%).

## Abbreviations

The following abbreviations are used in this manuscript:

AML	Acute myeloid leukemia
MRD	Minimal residual disease
AZA	Azacitidine
CR	Complete remission
PR	Partial remission
DP	Disease progression
ECOG	Eastern Cooperative Oncologic Group
ULN	Upper limit of normal
PFS	Progression-free survival
OS	Overall survival
