# Supplementary material for: Maintenance Treatment with 5-Azacitidine in Patients with Acute Myeloblastic Leukemia Ineligible for Intensive Treatment and with Response After Induction Chemotherapy: A Phase II Clinical Trial
Source: Cancers (Basel). 2025 Aug 18;17(16):2678. doi: 10.3390/cancers17162678 (PMC12384391; doi:10.3390/cancers17162678)
Supplement: Supplementary file 1 [file cancers-17-02678-s001.zip › cancers-3750018-supplementary.pdf]

cancers

MDPI

Supplementary materials for the submission "Maintenance Treatment with 5-Azacitidine in Patients with Acute Myeloblastic Leukemia Ineligible for Intensive Treatment and with Response After Induction Chemotherapy: A Phase II Clinical Trial" by Fernández et al.

Table S1. Dose adjustment of AZA treatment based on 1) blood count before the administration of AZA and 2) bone marrow cellularity and recovery time.

**Total neutrophil count ($x10^9$/L)**	**Platelets ($x10^9$/L)**	**% Dose of AZA in the next treatment administration**
<0.5	<25.0	50%
0.5-1.5	25.0-50.0	67%
>1.5	>50.0	100%
Bone marrow cellularity	% of the dose in the next cycle, if recovery is not achieved within 14 days	
	Recovery ≤ 21 days	Recovery > 21 days
15-50%	100%	50%
<15%	100%	33%

Table S2. IWG 2006 Response criteria, extracted from Komrokji et al.[19].

**Category**	**Response Criteria (must last at least 4 weeks)**
Complete Remission (CR)	Bone marrow: ≤5% myeloblasts with normal maturation of all cell lines<br>Persistent dysplasia will be noted<br>Peripheral blood: Hgb: ≥11 g/dL, platelets: ≥100 × 10^9/L, neutrophils: ≥1.0 × 10^9/L, blasts: 0%
Partial Remission	All CR criteria if abnormal before treatment except:<br>Bone marrow blasts decreased by ≥50% over pretreatment but still >5%<br>Cellularity and morphology not relevant
Marrow CR	Bone marrow: ≤5% myeloblasts and decrease by ≥50% over pretreatment<br>Peripheral blood: if HI responses, they will be noted in addition to marrow CR
Stable Disease	Failure to achieve at least PR, but no evidence of progression for >8 wks
Disease progression	For patients with:<br>Less than 5% blasts: 50% increase in blasts to 5% blasts<br>5%–10% blasts: 50% increase to 10% blasts<br>10%–20% blasts: 50% increase to 20% blasts<br>20%–30% blasts: 50% increase to 30% blasts<br>Any of the following:<br>At least 50% decrement from maximum remission/response in granulocytes or platelets<br>Reduction in Hgb by 2 g/dL<br>Transfusion dependence

CR, complete remission; PR, partial remission; wks, weeks.

Table S3. Baseline findings in the clinical history/comorbidities according to the MedDRA dictionary, *n (%).*

	**Total (n=32)**
Vascular disorders	19 (59.4)
Medical and surgical procedures	15 (46.9)
Metabolism and nutrition disorders	12 (37.5)
Benign, malignant, and unspecified neoplasms (including cysts and polyps)	9 (28.1)
Blood and lymphatic system disorders	7 (21.9)
Gastrointestinal disorders	7 (21.9)
Musculoskeletal and connective tissue disorders	7 (21.9)
Respiratory, thoracic, and mediastinal disorders	7 (21.9)
Cardiac disorders	6 (18.8)
Psychiatric disorders	6 (18.8)

 https://doi.org/10.3390/cancers17162678

Infections and infestations	5 (15.6)
Renal and urinary disorders	5 (15.6)
Reproductive sistema and breast disorders	4 (12.5)
Endocrine disorders	4 (12.5)
Immune system disorders	3 (9.4)
Skin and subcutaneous tissue disorders	2 (6.3)
Nervous system disorders	2 (6.3)
Hepatobiliary disorders	2 (6.3%)
Ocular disorders	2 (6.3)
Trauma, poisoning, and complications of medical and surgical procedures	1 (3.1)
Congenital, familial, and genetic disorders	1 (3.1)
General disorders and administration site conditions	1 (3.1)

*Patients could have more than one finding or concomitant disease.

Table S4. Description of adverse events presented by System Organ Class (SOC) and Preferred Term (PT)*, n (%).

			Severity**						
	**n (%) patients**	**N AE**	**G1**	**G2**	**G3**	**G4**	**Mi**	**Mo**	**S**
**N = 32**	31 (96.9)	308	134	94	34	12	19	11	4
**Blood and lymphatic system disorders**	25 (78.1)	131	30	40	33	11	5	10	2
Neutropenia	18 (56.3)	63	8	19	18	8	2	6	2
Anemia	11 (34.4)	21	9	7	3			2	
Thrombocytopenia	10 (31.3)	15	6	1	2	3	2	1	
Leucopenia	6 (18.8)	23	7	9	6		1		
Febrile neutropenia	4 (12.5)	6		1	4			1	
**Gastrointestinal disorders**	16 (50.0)	53	42	11					
Constipation	9 (28.1)	13	11	2					
Diarrhea	7 (21.9)	8	6	2					
Nausea	5 (15.6)	13	12	1					
Vomiting	4 (12.5)	4	3	1					
Pain in the upper abdomen	3 (9.4)	4	2	2					
Abdominal pain	2 (6.3)	2	1	1					
Hemorrhoids	2 (6.3)	2		2					
**Skin and subcutaneous tissue disorders**	13 (40.6)	29	14	13			2		
Pruritus	7 (21.9)	10	4	5			1		
Rash	3 (9.4)	10	5	5					
Erythema	2 (6.3)	2		1			1		
**General disorders and administration site conditions**	13 (40.6)	25	15	8			2		
Asthenia	7 (21.9)	11	4	6			1		
Peripheral edema	3 (9.4)	3	2				1		
Erythema at the site of administration	3 (9.4)	4	3	1					
Pyrexia	2 (6.3)	4	4						
**Infections and infestations**	11 (34.4)	18	5	11			2		
Respiratory tract infection	6 (18.8)	7	2	5					
Upper respiratory tract infection	2 (6.3)	2	1	1					
Urinary tract infection	2 (6.3)	2	1				1		
Infection related to a medical device	2 (6.3)	2		2					
**Metabolism and nutrition disorders**	8 (25.0)	25	12	3		1	6	1	2
Hyperuricemia	3 (9.4)	4	3			1			
Decreased appetite	2 (6.3)	6	3	3					
Hyperglycemia	2 (6.3)	5	1				4		
**Nervous system disorders**	5 (15.6)	10	7	3					
Headache	2 (6.3)	4	1	3					
Dizziness	2 (6.3)	3	3						
**Respiratory, thoracic, and mediastinal disorders**	4 (12.5)	4	3		1				
Cough	2 (6.3)	2	2						
**Musculoskeletal and connective tissue disorders**	3 (9.4)	7	3	4					
**Ear and labyrinth disorders**	2 (6.3)	2	1				1		
**Renal and urinary disorders**	2 (6.3)	2	1				1		

*Registered according to the Common Terminology Criteria for Adverse Events (CTCAE version 4.03) of the National Cancer Institute (NCI) and listed by the Preferred Term (PT) and the Organ/System Affected (SOC, System Organ Class) from the MedDRA dictionary (version 17.0),
**Two severity scales: G1-G5 and Mild (Mi)/Moderate (Mo)/Severe (S)/Life threatening/Death. G5 and Death are not included since no cases were reported.
Adverse events occurring in ≥ 5% of patients are included.
